# Acute myocardial infarction associated with thrombotic microangiopathy following a hump-nosed viper bite: a case report

**DOI:** 10.1186/s13256-017-1484-z

**Published:** 2017-10-30

**Authors:** Nipun Lakshitha de Silva, Lalindra Gooneratne, Eranga Wijewickrama

**Affiliations:** 1 University Medical Unit, National Hospital of Sri Lanka, Colombo, Sri Lanka; 2 Department of Pathology, Faculty of Medicine, University of Colombo, Colombo, Sri Lanka; 3 Department of Clinical Medicine, Faculty of Medicine, University of Colombo, 271, Kynsey road, Colombo 08, Sri Lanka

**Keywords:** Hump-nosed viper bite, Myocardial infarction, Thrombotic microangiopathy

## Abstract

**Background:**

Hump-nosed viper bite is the commonest cause of venomous snakebite in Sri Lanka. Despite initially being considered a moderately venomous snake more recent reports have revealed that it could cause significant systemic envenoming leading to coagulopathy and acute kidney injury. However, myocardial infarction was not reported except for a single case, which occurred immediately after the snakebite.

**Case presentation:**

A 50-year-old previously healthy Sri Lankan woman had a hump-nosed viper bite with no evidence of systemic envenoming during initial hospital stay. Five days later she presented with bite site cellulitis with hemorrhagic blisters, acute kidney injury, and evidence of microangiopathic hemolytic anemia and thrombocytopenia with normal coagulation studies. She was managed with supportive care that included intravenously administered antibiotics, blood transfusions, and hemodialysis; both her microangiopathic hemolytic anemia and thrombocytopenia improved without any specific intervention. On day 10 she developed: a non-ST elevation myocardial infarction complicated with acute left ventricular failure evidenced by acute shortness of breath with desaturation despite adequate ultrafiltration; new onset lateral lead T inversions in electrocardiogram; raised troponin I titer; and hypokinetic segments on echocardiogram. She was managed with low molecular weight heparin and antiplatelet drugs, which were later discontinued due to upper gastrointestinal bleeding. Her hospital stay was further complicated by hospital-acquired pneumonia and deep vein thrombosis involving her ileofemoral vein. She died on day 33 from the snakebite.

**Conclusions:**

Myocardial infarction after snakebites is rarely reported. This is the first case report of a patient developing a myocardial infarction during the recovery phase of thrombotic microangiopathy following a hump-nosed viper bite. The possibility of thrombotic risk related to thrombotic microangiopathy following hump-nosed viper bite is an area that is poorly studied; it needs further attention.

## Background

Hump-nosed vipers of the genus *Hypnale* consist of three species *Hypnale hypnale* (found in Sri Lanka and parts of India), *Hypnale zara*, and *Hypnale nepa* (endemic to Sri Lanka) [1]. Hump-nosed viper bite is recognized as the commonest cause of venomous snakebite in Sri Lanka [2] and according to studies where species identification was done the most common species is *Hypnale hypnale* [3, 4].

Clinical manifestations of local envenoming predominate during the early phase; the clinical manifestations include pain, swelling, tissue necrosis, and hemorrhagic blisters [4]. The commonest systemic manifestations include coagulopathy evidenced by spontaneous bleeding [3] and subclinical coagulopathy evidenced by altered coagulation profile [5] or features of thrombotic microangiopathy (TMA) [6]. Although the early reports from case series of patients with hump-nosed viper bites revealed a lack of systemic effects other than coagulopathy [7], it is now clearly recognized that hump-nosed viper bites can lead to a multitude of systemic envenoming features and even death [5]. Acute kidney injury was noted in up to 20% of cases in recent studies [5]. Neurological and cardiac effects are not well recognized in patients with hump-nosed viper bites and cardiac effects have been limited to transient electrocardiogram (ECG) changes according to the available literature [5].

There is a case report of acute myocardial infarction which occurred within half an hour of envenoming by hump-nosed viper bite in association with TMA [8]. We report a case where a previously healthy woman with no major cardiovascular risk factors developed a myocardial infarction during the recovery phase of TMA from a hump-nosed viper bite.

## Case presentation

A 50-year-old previously well Sri Lankan woman presented to a local hospital following a bite on her right foot by a snake. The snake was killed and brought to the hospital by members of her family and was confirmed as a hump-nosed viper by the attending doctors. Species identification was not performed. She only had features of local envenoming including pain, local swelling, and blistering. She did not have any bleeding manifestations, maintained good urine output, and there were no neurological deficits. Her full blood count, serum creatinine, and 20-minute whole blood clotting test were normal on the first day. She was given paracetamol, orally administered antibiotics, and observed in the local hospital and was discharged on the second day of admission.

Five days after the snakebite she was readmitted to the local hospital due to worsening swelling and pain of her right foot and leg. She was afebrile on admission and did not complain of any reduction in urine output or bleeding manifestation. However, by the next day her urine output was found to be low and she was transferred to the National Hospital of Sri Lanka (NHSL) for further management. On admission to NHSL she was fully conscious and only complained of pain and swelling of right lower limb, reduced urine output, and mild shortness of breath at rest. She was pale, not icteric and there was generalized edema. There was right lower limb cellulitis with hemorrhagic blisters. There was bruising on puncture sites. She was dyspneic with a respiratory rate of 24 breaths/minute; her heart rate was 100 beats/minute and blood pressure was 120/80 mmHg. Oxygen saturation on air was 96%. Her jugular venous pressure was raised. A precordial examination was normal. A respiratory examination revealed bibasal crackles suggestive of pulmonary edema. An abdominal examination was normal. She had a urethral catheter with no urine.

Laboratory investigations on admission revealed a hemoglobin of 7 g/dl, mean corpuscular volume of (MCV) 82.6 fl, and red cell count of 2.36 × 10^12^/l. Her white cell count (WCC) was 10.96 × 10^9^/l with 75% neutrophils and 17% lymphocytes. Her platelet count was 92 × 10^9^/l. Her blood picture revealed fragmented red cells suggestive of microangiopathic hemolytic anemia (MAHA) and toxic neutrophils suggestive of bacterial infection. Her serum lactate dehydrogenase (LDH) level was 2300 U/L. Her prothrombin time was 12.6 seconds and activated partial thromboplastin time (APTT) was 24 seconds. Serum creatinine was 888 μmol/l, while potassium was 7 mmol/l and sodium was 126 mmol/l. Venous blood gas revealed a pH of 7.29 with bicarbonate of 11 mmol/l. A clinical diagnosis of a hump-nosed viper bite leading to cellulitis, acute kidney injury, and TMA was made. Her ECG on admission was normal.

She was started on intravenously administered meropenem and metronidazole and urgent hemodialysis was arranged via a femoral catheter. In the absence of evidence for the benefit of therapeutic plasma exchange or fresh frozen plasma in MAHA following hump-nosed viper bites it was decided to manage her without these therapeutic modalities. She underwent regular hemodialysis with adequate ultrafiltration on the sixth, seventh and ninth days after the snakebite. She remained anuric throughout. Her thrombocytopenia improved within the next 2 days with her platelet count rising to 160 × 10^9^/l and some fragmented red cells remained in the blood picture. She was given two packs of red cell concentrates over the next 2 days.

**Fig. 1:**
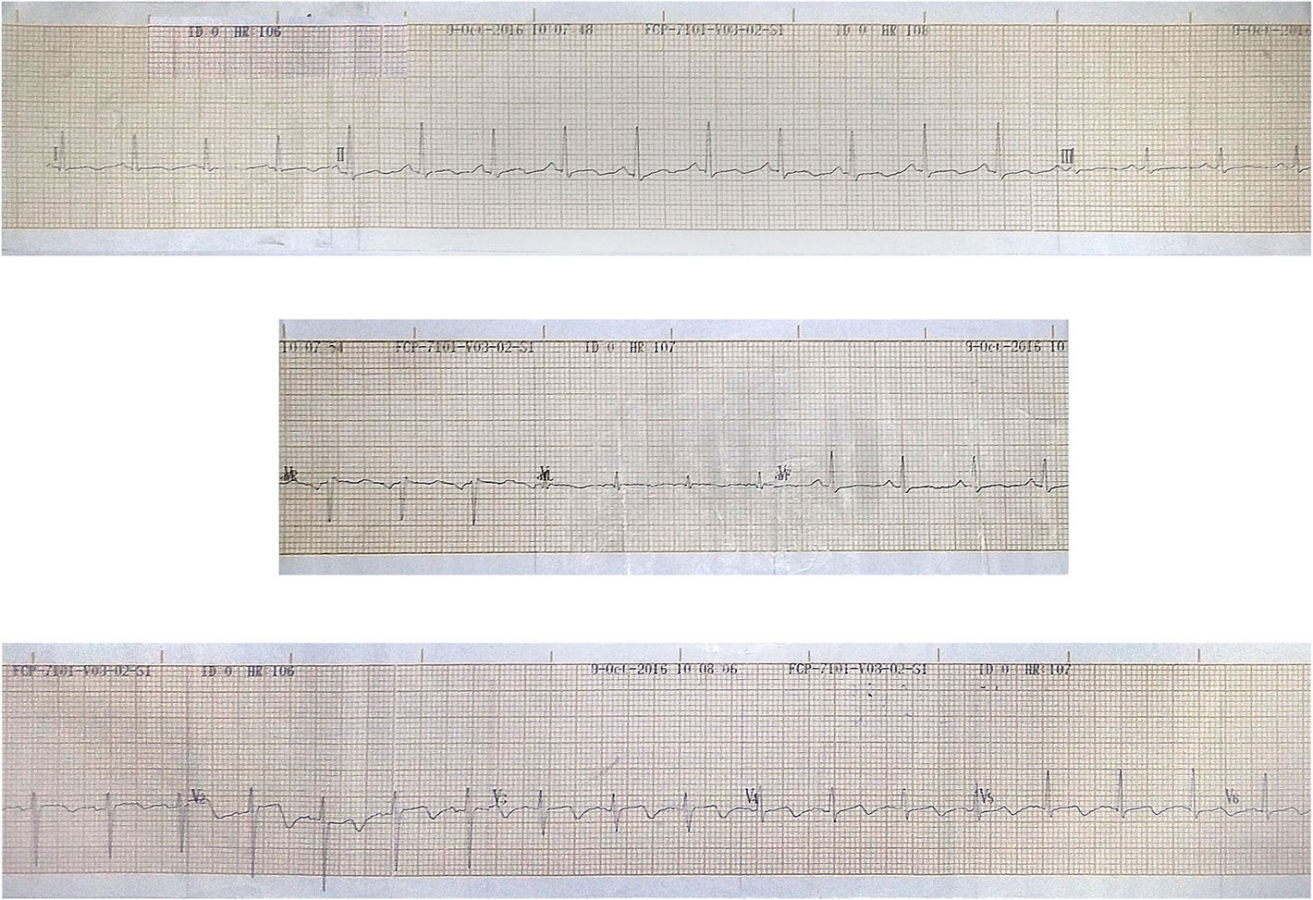
Electrocardiogram taken during non-ST elevation myocardial infarction. This was taken soon after the onset of acute retrosternal chest pain and shortness of breath with evidence of pulmonary edema. It shows T wave inversion in V3 to V6 and aVL which were not present in the electrocardiogram taken on admission

**Fig. 2:**
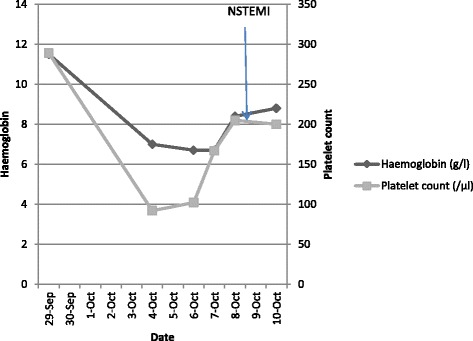
Trend of hemoglobin and platelet count during the initial period after snakebite. Soon after the snakebite, our patient’s hemoglobin and platelet count were normal. Thereafter there is a period with no record of our patient’s hemoglobin level and platelet count because she was discharged initially from a local hospital. She then developed thrombocytopenia and anemia due to thrombotic microangiopathy. Later, her thrombocytopenia starts to recover spontaneously. Anemia was corrected using red cell transfusion. However, serum lactate dehydrogenase was high and schistocytes were persistent in blood picture. She developed acute coronary syndrome while thrombocytopenia was resolving but thrombotic microangiopathy persisted. *NSTEMI* non-ST elevation myocardial infarction

On day 10 she developed acute onset shortness of breath and retrosternal chest pain with bibasal crackles on lung auscultation despite adequate ultrafiltration the previous night and adherence to a strict fluid restriction. Her oxygen saturation on air was 89% and improved to 97% with 5% oxygen via facemask. An ECG showed T wave inversion in V3 to V6 and aVL leads (Fig. 1). Serum troponin I titer was 0.433 ng/ml (normal < 0.03) 12 hours after the onset of chest pain. A diagnosis of non-ST elevation myocardial infarction was made. At that time her hemoglobin was 8 g/dl, platelet count was 205 × 10^9^/l, and serum LDH was 2100 U/l (Fig. 2). Her serum electrolytes were within normal range. She was started on subcutaneous enoxaparin 60 mg/day, aspirin 75 mg/day, clopidogrel 75 mg/day, and atorvastatin 20 mg/day. Any existing coagulopathy was excluded by thromboelastography before initiating the antiplatelet drugs and anticoagulation. She was given a few more packed cell transfusions during hemodialysis. An echocardiogram done 3 days later revealed anterior and lateral wall hypokinesia in the left ventricle and her ejection fraction was at 45 to 50%.

On day 15 she developed an upper gastrointestinal bleed confirmed by an esophagogastroduodenoscopy. Antiplatelet drugs and anticoagulation were withheld and she was transfused with blood.

**Fig. 3:**
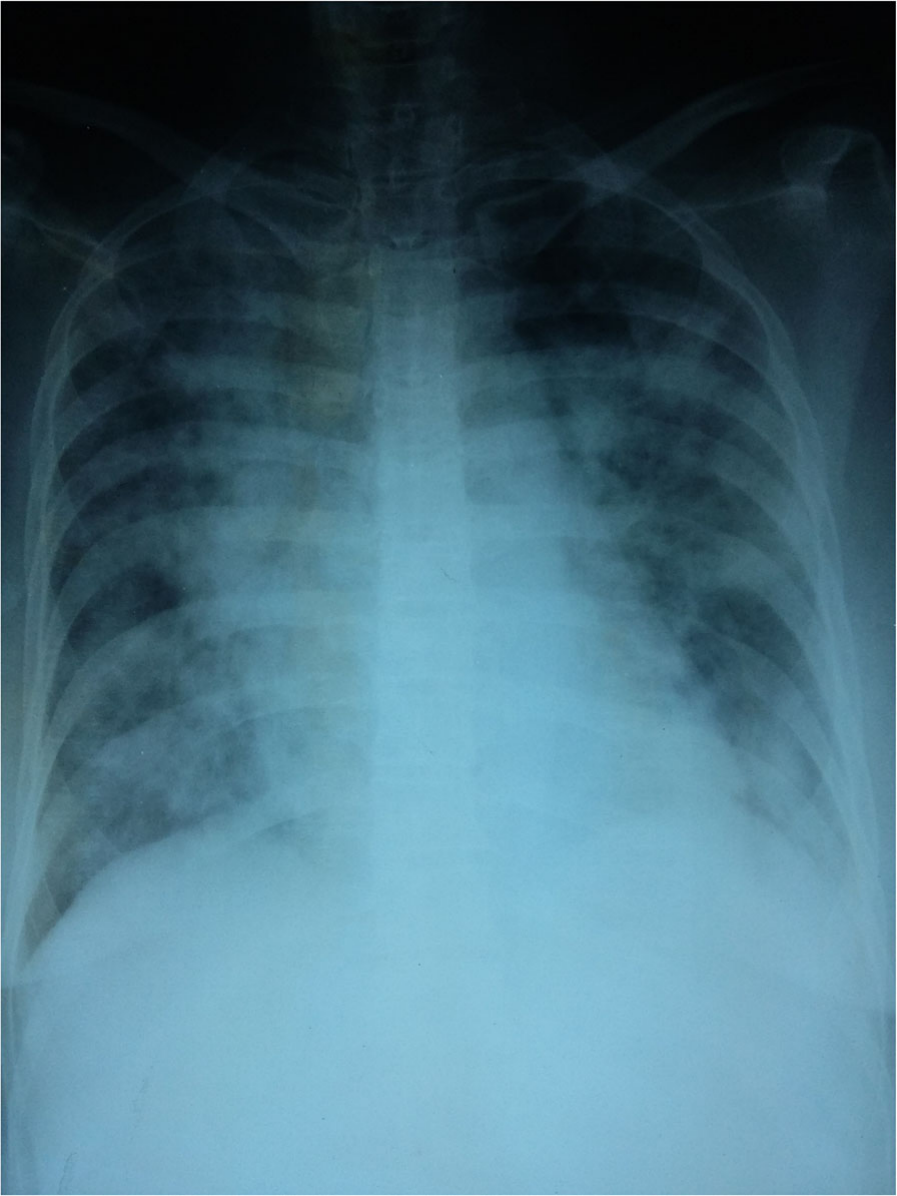
Chest radiograph which was taken when our patient deteriorated on day 18. This chest radiogram was taken on day 18 after the snakebite when our patient developed worsening shortness of breath with fever. There are bilateral diffuse opacifications in all lung fields. In the clinical context with shortness of breath, fever, prolonged hospital stay, and bronchoalveolar lavage and endotracheal tube secretion positivity for coliforms this appearance was attributed to hospital-acquired bronchopneumonia

On day 18 she developed worsening shortness of breath associated with reduced oxygen saturation and fever. A repeat ECG did not show any new changes. A chest radiograph revealed bilateral diffuse opacification (Fig. 3) which persisted even after adequate ultrafiltration. A presumptive diagnosis of hospital-acquired bronchopneumonia was made and her antibiotics were changed to piperacillin-tazobactam and teicoplanin. Subsequently she developed respiratory failure and was intubated and transferred to our Medical Intensive Care Unit (MICU) for ventilator support. Bronchoscopy revealed absence of pulmonary hemorrhage and both bronchoalveolar lavage fluid and tracheal aspirate grew coliforms resistant to all available antibiotics in the hospital setting and sensitive only to colistin which was not available. Her stay in MICU was further complicated with a deep vein thrombosis involving her right ileofemoral vein which was treated with unfractionated heparin while maintaining APTT within the therapeutic range. She progressively deteriorated and developed a cardiac arrest and died on day 33 of the snakebite (Table 1).

**Table 1:** Timeline of events with events and interventions

Date	Events	Diagnostic tests	Interventions
29/09/2016	Hump-nosed viper bite, admission to local hospital		
30/09/2016		Full blood count, serum creatinine, 20-minute whole blood clotting test – normal	Discharged on orally administered analgesics and antibiotics
03/10/2016	Worsening swelling and pain in bite site due to cellulitis, readmitted to local hospital		Started on intravenously administered antibiotics
04/10/2016	Reduced urine output, transferred to National Hospital of Sri Lanka. Mild shortness of breath, pallor, edema, pulmonary edema	Hemoglobin 7 g/dl, platelets 92,000. Blood picture – microangiopathic hemolytic anemia, LDH 2300 U/l, creatinine 888 μmol/l, ECG – normal	Intravenously administered antibiotics. Hemodialysis with red cell transfusion
05/10/2016 to 07/10/2016	Clinical improvement, resolution of shortness of breath	Platelets 160,000, persistent red cell fragments	Regular hemodialysis
08/10/2016	Acute onset shortness of breath, retrosternal tightening chest pain, and bibasal crackles in both lung fields	ECG – T wave inversion in V3 to V6 and aVL, troponin positive, platelets 205,000, LDH 2100 U/l. Echocardiogram (11/10/2016) – lateral wall dyskinesia	Anticoagulation with enoxaparin renal-adjusted doses, antiplatelet drugs and statins
13/10/2016	Melena	Esophagogastroduodenoscopy, acute bleeding in duodenum	Withheld antiplatelet drugs and anticoagulation, red cell transfusion, omeprazole infusion
16/10/2016	Worsening shortness of breath, fever, respiratory failure	Chest radiogram – bilateral diffuse opacification. Bronchoalveolar lavage – multi-resistant coliforms	Intravenously administered antibiotic changed. Intubation and ventilation with ICU care
24/10/2016	Unilateral limb swelling	Duplex scan of lower limb – ileofemoral deep vein thrombosis	Unfractionated heparin infusion
31/10/2016	Death		

*ECG* electrocardiogram, *ICU* Intensive Care Unit, *LDH* lactate dehydrogenase

## Discussion

Hump-nosed viper, which was considered a moderately venomous snake in the past, is now increasingly recognized as a snake that can cause considerable morbidity, sometimes leading even to death. Hematological and renal toxicity constitute the two commonest systemic effects of hump-nosed viper bite [5]. Cardiac manifestations are minimal apart from transient ECG changes attributed mainly to hemodynamic and electrolyte disturbances that occur due to envenoming. A study done in 1999 which included 32 patients did not reveal any cases of cardiac muscle injury manifested as a rise in serum troponin titer [9].

Myocardial infarction following viper bites has been reported in the literature with the majority occurring within a few hours after the bite [10, 11]. This holds true for the one reported case of myocardial infarction following hump-nosed viper bite where the infarction occurred soon after the bite prior to arrival at a hospital [8]. Presumed mechanisms include hypovolemic shock, coronary artery spasm, and hypercoagulability due to snake venom [10]. Pre-existing atheromatous disease may also have contributed. Strokes have also been reported following viper bites presumably due to a similar mechanism [12, 13]. There is one case report of a patient who developed ischemic stroke after a hump-nosed viper bite [14].

Cardiac and cerebral ischemic episodes are less well reported following bites by the Sri Lankan Russell’s viper probably related to the differences in the composition of the venom [9]. Even the available cases are examples of early complications of snakebite [15].

In our patient the diagnosis of acute myocardial infarction was supported by the history of acute onset retrosternal chest pain, electrocardiographic changes, and the raised troponin I titer. Changes in troponin titers need to be interpreted with caution in the presence of renal impairment since abnormal renal function per se could lead to a raised titer. A rising titer could have been more supportive of a myocardial infarction; however, this could not be performed in our patient due to logistical issues. The presence of regional wall motion abnormalities on an echocardiogram corresponding to electrocardiographic changes was also supportive of a myocardial infarction [16]. A coronary angiogram was not performed in the presence of acute kidney injury to avoid added nephrotoxicity secondary to contrast exposure. Cardiac sequelae in TMA are related to microvascular thrombosis rather than major coronary artery occlusion according to pathologically proven evidence [17]. This further weakens the indication for coronary angiography unless the objective is to look for pre-existing atheromatous disease.

The mechanism for the acute coronary syndrome in this patient is difficult to define due to the lack of understanding of acute organ ischemia following viper bites. However, several key possibilities can be worked out with the available knowledge. In this middle-aged woman the possibility of pre-existing atheromatous disease cannot be ignored; however, the fact that she was free from common major risk factors including diabetes mellitus, hypertension, hyperlipidemia, and tobacco smoking has to be borne in mind. During the time that our patient developed myocardial infarction her blood pressure was normal with no hemodynamic instability. There were no episodes of hypotension during the dialysis performed the previous day. Her serum electrolytes done on the same day were also normal. Therefore hemodynamic instability or electrolyte disturbances are unlikely to be the cause of the ECG changes observed in this patient.

The venom of the hump-nosed viper is known to have procoagulant as well as anticoagulant properties that can lead to thrombosis as well as venom-induced consumptive coagulopathy (VICC) in its victims. This depends on the rapidity of clot formation and degradation [18]. However, in our patient, the timing of the thrombotic event does not favor a direct effect of the snake venom. Following a period of VICC some patients tend to develop TMA with normal coagulation profile and absent bleeding manifestations [19]. This might have close similarities to thrombotic thrombocytopenic purpura (TTP)-like syndrome clinically; however, there is no evidence to label this entity as a secondary TTP [6]. The literature neither provides information about the clinical and biochemical nature of this condition nor any evidence-based recommendations on the management. There are reports that TMA following snakebites does resolve spontaneously unlike TTP which has a very poor outcome in the absence of plasma exchange [19].

Clinically and pathologically proven myocardial infarctions have been reported in patients with TTP [20, 21] and other forms of TMA [22]. High levels of serum LDH is known to be associated with increased risk of acute myocardial infarction in TMA [22]. In patients with TTP the risk of thrombosis is highest during the recovery phase of thrombocytopenia and initiation of antiplatelet drugs is recommended when their platelet count rises above 50 × 10^9^/l [23]. The patient under discussion developed myocardial infarction 5 days after the onset of TMA at which point her thrombocytopenia was actually resolving. Therefore it is reasonable to assume that the TMA was the most likely underlying cause for the myocardial infarction in this patient.

There has been a case of acute myocardial infarction following a hump-nosed viper bite reported previously where the patient developed the myocardial infarction within hours after the bite [8]. Although the patient concerned had thrombocytopenia and early MAHA with few schistocytes on blood picture at the time of the event, there was no significant drop in the level of hemoglobin to suggest a full-blown TMA. The patient had abnormal coagulation tests indicating presence of VICC at the time suggesting possible venom-induced procoagulant activity contributing to the coronary artery thrombosis. In comparison, our patient developed the myocardial infarction 10 days after the bite at which point there was no evidence of VICC with normal coagulation tests. Our patient had features of full-blown TMA as evidenced by thrombocytopenia, MAHA on blood picture, significant drop in hemoglobin, and high LDH prior to the development of myocardial infarction. Therefore it is reasonable to assume that TMA was the cause for myocardial infarction in the present case whereas the cause for the previously published case was not conclusive. Hence, our case could be considered the first authentic case of hump-nosed viper bite leading to development of myocardial infarction as a result of TMA.

In the absence of a thorough understanding of the pathophysiology of the disease there is no guidance on the prevention and treatment of such ischemic complications related to snakebite. We initiated our patient on anticoagulants and antiplatelet drugs in keeping with the standards of care in the management of acute coronary syndrome. Unfortunately she developed an acute gastrointestinal bleed soon after, despite having a normal platelet count and coagulation at the time. This led to a longer hospital stay, which eventually resulted in her developing a multi-resistant hospital-acquired pneumonia resulting in respiratory failure and death.

## Conclusions

Our experience in managing this patient provides the first case report of a myocardial infarction in a patient who was recovering from TMA following a hump-nosed viper bite. The exact mechanism of TMA in viper envenomation and its role in causing coronary ischemia are poorly understood at present and further in-depth studies are required to recommend optimal preventative and treatment strategies for the management of TMA following hump-nosed viper bites.

## Abbreviations

APTT: Activated partial thromboplastin time
ECG: Electrocardiogram
LDH: Lactate dehydrogenase
MAHA: Microangiopathic hemolytic anemia
MCV: Mean corpuscular volume
MICU: Medical Intensive Care Unit
NHSL: National Hospital of Sri Lanka
TMA: Thrombotic microangiopathy
TTP: Thrombotic thrombocytopenic purpura
VICC: Venom-induced consumptive coagulopathy
WCC: White cell count

## Authors’ information

Dr Nipun Lakshitha de Silva: Postgraduate trainee in clinical medicine at University Medical Unit, National Hospital of Sri Lanka and lecturer (probationary) in Clinical Medicine, Faculty of Medicine, General Sir John Kotelawala Defence University. Lalindra Gooneratne: Senior Lecturer in pathology and consultant hematologist, Department of Pathology, Faculty of Medicine, University of Colombo. Eranga Wijewickrama: Specialist nephrologist, University Medical Unit, National Hospital of Sri Lanka and Senior Lecturer in Clinical Medicine, Department of Clinical Medicine, Faculty of Medicine, University of Colombo.
